# Noise-Induced Tinnitus Using Individualized Gap Detection Analysis and Its Relationship with Hyperacusis, Anxiety, and Spatial Cognition

**DOI:** 10.1371/journal.pone.0075011

**Published:** 2013-09-12

**Authors:** Edward Pace, Jinsheng Zhang

**Affiliations:** 1 Department of Otolaryngology-Head and Neck Surgery, Wayne State University School of Medicine, Detroit, Michigan, United States of America; 2 Department of Communication Sciences & Disorders, Wayne State University College of Liberal Arts & Sciences, Detroit, Michigan, United States of America; University of Salamanca- Institute for Neuroscience of Castille and Leon and Medical School, Spain

## Abstract

Tinnitus has a complex etiology that involves auditory and non-auditory factors and may be accompanied by hyperacusis, anxiety and cognitive changes. Thus far, investigations of the interrelationship between tinnitus and auditory and non-auditory impairment have yielded conflicting results. To further address this issue, we noise exposed rats and assessed them for tinnitus using a gap detection behavioral paradigm combined with statistically-driven analysis to diagnose tinnitus in individual rats. We also tested rats for hearing detection, responsivity, and loss using prepulse inhibition and auditory brainstem response, and for spatial cognition and anxiety using Morris water maze and elevated plus maze. We found that our tinnitus diagnosis method reliably separated noise-exposed rats into tinnitus^(+)^ and tinnitus^(−)^ groups and detected no evidence of tinnitus in tinnitus^(−)^ and control rats. In addition, the tinnitus^(+)^ group demonstrated enhanced startle amplitude, indicating hyperacusis-like behavior. Despite these results, neither tinnitus, hyperacusis nor hearing loss yielded any significant effects on spatial learning and memory or anxiety, though a majority of rats with the highest anxiety levels had tinnitus. These findings showed that we were able to develop a clinically relevant tinnitus^(+)^ group and that our diagnosis method is sound. At the same time, like clinical studies, we found that tinnitus does not always result in cognitive-emotional dysfunction, although tinnitus may predispose subjects to certain impairment like anxiety. Other behavioral assessments may be needed to further define the relationship between tinnitus and anxiety, cognitive deficits, and other impairments.

## Introduction

Tinnitus is a sound perception that occurs within the ear or head in the absence of an external source. An estimated 50 million Americans experience tinnitus, with 16 million seeking medical intervention [1], [2]. Tinnitus sufferers frequently struggle with difficult sleeping [3]–[5], irritability [6], [7], and cognitive deficits [8]–[12], and are at greater risk for depression, anxiety [13]–[15], and in some cases suicide [16]. From this, it is evident that the emotional and cognitive effects of tinnitus have a significant impact on patients and merit thorough investigation.

In support of the cognitive-emotional impact of tinnitus, a growing volume of literature shows that related limbic structures, including the hippocampus and amygdala, may be activated during tinnitus perception. For example, the hippocampus may be activated in tinnitus patients as revealed by increased regional cerebral blood flow [17], [18]. Studies in rats have demonstrated that intense sound exposure previously shown to induce tinnitus alters responses of hippocampal place cells [19] and impairs hippocampal neurogenesis [20]. The amygdala may also be activated, as evidenced by elevated regional cerebral blood flow [17] and increased fos-like immunoreactivity following sound exposure and salicylate injections, which are known tinnitus-inducers [21]–[23]. Higher cortisol levels and blunted cortisol response to stress have also been found in tinnitus patients and are indicative of hypothalamic pituitary adrenal axis activation, which is mediated by the amygdala and the hippocampus [24], [25].

Despite evidence linking limbic functioning and tinnitus perception [17], [26]–[28], the relationship between tinnitus, limbic-associated functioning, and the underlying mechanisms remains unclear. For example, tinnitus patients performed worse than control subjects on verbal fluency and reading span tests, indicating deficits in working memory [12]. Other memory assessments however, including tests of serial and spatial recall and five-choice serial reaction time with a dual task for memory, have found no significant difference between tinnitus subjects and controls [10], [29]. Additionally, not all individuals with tinnitus have anxiety [30]. In animal studies, rats that developed tinnitus following acoustic trauma showed no impairment in spatial learning and memory [31] and no significant increase in anxiety level [32], though they did show compromised impulse control and social interaction [32], [33]. Tinnitus, therefore, has a complex relationship with cognitive-behavioral functioning in both humans and animals.

Amidst the conflicting data, the presence of confounds is a major complicating factor in the relationship between tinnitus, cognition, and anxiety. One potential confound can arise when tinnitus is induced by noise exposure, which is one of the most prominent causes of tinnitus and can itself induce anxiety when presented on a regular basis [34], [35]. Hearing loss also frequently accompanies tinnitus, and while some evidence suggests that tinnitus perception is more anxiogenic than hearing loss, hearing loss may provoke anxiety in some individuals [36], [37]. Hyperacusis reportedly co-occurs in between 40 to 80% of tinnitus patients [38]–[40] and results in patients suffering distress from common sounds, and in some cases, social withdrawal [41]. In addition, tinnitus sufferers frequently struggle with comorbid depression and anxiety [42]–[47]. This obfuscates the relationship between tinnitus and limbic-associated functioning since depression and anxiety not only affect emotional processing and alter neural activity in limbic structures [48]–[51], but can also hinder cognitive functioning, including memory [52], [53].

To help address the clinical challenges posed by tinnitus, animal models of noise-induced tinnitus have been utilized [54]–[67], and some studies have begun examining its cognitive and emotional correlates [31]–[33], [68]. A critical task in this area, however, is diagnosing tinnitus. Due to the number of different tinnitus animal models, it can be difficult to compare tinnitus correlates across studies. While gap-detection of the acoustic startle reflex has seen prominent use over the past several years [20], [58], [60], [61], [63], [64], [66], [67], [69]–[76], it presently lacks standardized tinnitus diagnosis. Many studies using gap-detection have not formally divided animals into tinnitus^(+)^ and tinnitus^(−)^ groups [60], [61], [63], [70], [72], [73], [75], [76], which is a critical step since not all individuals exposed to acoustic trauma develop tinnitus. Additionally, those that have divided animals based on tinnitus perception are still lacking a common, statistically-driven method [20], [64], [66], [67], [71]. Increased commonality of tinnitus diagnosis and a rigorous method to identify noise-exposed tinnitus positive rats is needed to improve solidarity between various reports and to better approach clinical challenges, such as the complicated relationship between noise-induced tinnitus, related audiological impairment, and cognition and emotion.

In the current study, we conducted experiments using Long-Evans rats and investigated the effect of intense tone-induced tinnitus, hearing loss, and hyperacusis-like behavior on spatial learning and memory, and anxiety. Rats were individually diagnosed with tinnitus using a gap detection paradigm and tested for hearing loss, detection and responsivity using auditory brainstem responses (ABRs) and prepulse inhibition (PPI). Rats were then tested for spatial learning and memory with the Morris water maze (MWM) and for anxiety with the elevated plus maze (EPM). Our results showed that our tinnitus diagnosis method was reliable and that the tinnitus^(+)^ group also exhibited hyperacusis-like behavior, although tinnitus exerted no significant effect on cognition or anxiety at the group level.

## Materials and Methods

### Animal Subjects

Twenty-nine male Long-Evans rats (60–70 days-old) were purchased from Charles River Laboratories. Eighteen rats were exposed twice to an intense tone and were divided into tinnitus^(+)^ and tinnitus^(−)^ groups, depending on tinnitus development following exposure. Four rats exhibited poor acoustic startle reflex performance and were excluded from the study prior to tone exposure. A group of seven age-matched and unexposed rats served as controls. All procedures were approved by the Institutional Animal Care and Use Committee at Wayne State University and were in accordance with the regulations of the Federal Animal Welfare Act.

### Gap detection (GAP) and Prepulse Inhibition (PPI) Testing - Before Tone Exposure

Rats underwent behavioral testing for tinnitus and auditory detection using GAP and PPI, as previously described [64]. All behavioral testing was conducted inside a sound-attenuation booth with acoustic startle reflex hardware and software (Kinder Scientific, Poway, CA). In the GAP procedure, each rat was presented with constant, 60 dB SPL background noise consisting of bandpass signals centered at 6–8, 10–12, 14–16, or 26–28 kHz, or broadband noise (BBN). A 115 dB SPL, 50 ms noise burst served as the startle stimulus to induce the acoustic startle reflex. During the background noise, the rat was either presented with the startle stimulus alone (startle only condition) or the startle stimulus preceded by a silent gap embedded within the background noise (GAP condition). Silent gaps were 40 ms in duration with a lead interval of 90 ms to the startle stimulus. The startle reflex of rats was measured in response to 3 conditions: 1) background noise alone, 2) startle only, and 3) GAP. Four trials of the background noise alone condition and 8 trials for the startle only and GAP condition each were administered for every background noise frequency and BBN.

The PPI procedure was the same as gap-detection except that no background noise or gaps were used. Rats were either presented with the startle stimulus alone (startle only condition) or the startle stimulus preceded by a prepulse (PPI condition). Prepulses were 40 ms in duration with a lead interval of 90 ms and were presented at 60 dB SPL. The startle reflex of rats was measured in response to 2 conditions: 1) startle only, and 2) PPI. Eight trials for both the startle only and PPI conditions were administered for each prepulse frequency and BBN prepulses.

Both GAP and PPI were run sequentially with a 2 min acclimatization period before each test. Two trials of the startle stimulus without background noise were presented after the acclimatization period to trigger any initial, exaggerated startle reflexes, and were not used in analysis. Startle-only and GAP or PPI conditions were arranged in a pseudorandom sequence to prevent order effects. The running time for both tests was approximately 1 hour and 40 min. In order to achieve stable baseline behavioral data, rats were tested an average of 10 times over a month period.

### Auditory Brainstem Responses (ABRs) - Before Tone Exposure

Each rat underwent click and tone-burst ABR to assess hearing thresholds. Anesthesia was induced through inhalation of mixed air (0.6 L/min) and isoflurane (5%, v/v) and was reduced to 0.4 L/min and 2–3% v/v for maintenance during testing. ABR responses were elicited by click and tone burst stimuli (10 ms duration, 0.5 ms rise/fall) delivered from a TDT EC1 model electrostatic speaker (Tucker Davis Technologies, Alachua, FL) through a tube inserted into the external auditory canal. Stimuli were generated by a TDT RX6 multifunction processor, calibrated with a microphone (Model 4016, ACO Pacific) and SigCalRP® software, and presented from 80 dB peak equivalent SPL down to 5 dB in 5 dB decrements.

Evoked potentials were recorded using subdermal platinum-coated tungsten needle electrodes. The positive recording electrode was placed at the vertex, while the reference electrode was placed below the ear pinna ipsilateral to the speaker, and the ground electrode was placed below the contralateral ear pinna. ABR responses were amplified, bandpass-filtered at 300 to 3000 Hz, and notch-filtered at 60 Hz. Responses to click and tone-burst stimuli were averaged 300 and 400 times, respectively. The sampling rate for data acquisition was 50 kHz. Experimental operation was controlled by SigGenRP® and BioSigRP® TDT software installed in an IBM terminal connected to a System 3 TDT workstation. For analysis, ABR threshold was considered the lowest intensity at which a distinct portion of the biological waveform remained.

### Intense Tone Exposure to Induce Tinnitus

After stable baseline GAP and PPI data were observed, rats were unilaterally exposed (left ear) to a 118–120 dB peak SPL, 10 kHz tone for 2 hours. Unilateral exposure was conducted so that at least one normal hearing ear remained, which has been shown to be sufficient for normal gap-detection [63]. Five weeks later, a second exposure was conducted for 3 hours and served to reinforce or enhance the previously induced tinnitus. The second exposure also increased relevance to clinical cases, where individuals have often incurred acoustic trauma more than once. For further clinical significance and to circumvent protective effects of anesthesia [71], [77], [78], rats were exposed while awake.

Prior to exposure, each rat was anesthetized through inhalation of mixed air (0.6 L/min) and isoflurane (5%, v/v). The right ear canal was then occluded with an earplug followed by application of mineral oil to seal any additional open spaces. The plugged ear was sutured shut to keep the plug in place. Each rat was given 30 min to recover from anesthesia. Between 4 to 6 rats were placed in a cage and exposed at a time. The exposure tone was presented through a TW67 speaker (Pyramid Car Audio, Brooklyn, N.Y.) placed face-down on top of a 44×23×22 cm polycarbonate rat cage with floor bedding consisting of wood shavings, and was calibrated at the estimated average position of the rats (Bruel & Kjar, BZ-7100). The tone was produced by a TDT multifunction processor and amplified through an RA 300 amplifier (Alesis, Cumberland RI). Operation was controlled by a custom Constant Tone OpenEx program (TDT). Following tone exposure, rats were again anesthetized and their earplugs and sutures were removed. The control group underwent the same procedures except that no tone was delivered. Some rats tended to orient their heads toward the speaker during the initial minutes of the tone exposure, however, rats remained relatively still throughout the majority of the exposure so this did not appear to be problematic.

### GAP, PPI, and ABR Testing – After Tone Exposure

Behavioral testing and ABRs were conducted using the same parameters as before tone exposure. Rats were tested behaviorally one day after exposure and on a biweekly basis until MWM and EPM testing (6 weeks after exposure). ABR testing was administered at 1 and 8 weeks post-exposure.

### Morris Water Maze (MWM)

As described elsewhere [79], a one-day water maze procedure was carried out 6 weeks after exposure to assess spatial learning and memory. Testing was conducted in a fiberglass pool (183 cm in diameter) filled with water opacified with white, non-toxic paint. For analytical purposes, the interior of the pool was virtually divided into 4 zones of equal size. An escape platform 11 cm in diameter was hidden in the middle of zone 4 (target zone) at 2 cm below the surface level of the water.

Rats were given a total of 4 trials. Each trial was initiated from one of 4 random starting points by lowering a rat into the water while facing the pool wall. If a rat failed to locate the escape platform within 90 seconds, it was taken from the water and placed on the platform for 3 seconds. Following the last escape trial, the platform was removed and a probe trial was administered where a rat was allowed to swim freely for 90 seconds. The rat was dried with a cloth towel and placed back inside its cage after each trial. Rats were analyzed on time required to locate the escape platform (escape latency), swimming velocity, and time spent and entries into the target zone (probe trial). Data were acquired using Ethovision XT (Noldus Information Technology, Wageningen, Netherlands), a video tracking and analysis software.

### Elevated Plus Maze (EPM)

Rats were tested on the EPM 6 weeks after exposure to measure anxiety level. The plus maze was constructed from wood and made into a cross shape with two opposing open arms (28 cm×10 cm) and two opposing closed arms (47.5 cm×10 cm×39 cm) extending from a center square (10 cm×10 cm). The maze was elevated approximately 60 cm above the ground. Duct tape was applied to the maze surface where rats would walk. A rat was placed in the center square facing an open arm and allowed to explore the maze for 5 min. Rats were observed by a hidden experimenter and scored on the number of entries into the open and closed arms and time spent in the open arms. An entry was defined as setting all four paws in an arm. The maze was cleaned, deodorized and dried between each test with 70% ethanol.

### Data Analysis

All gap-detection startle force data were divided by the mean of the corresponding startle only responses and converted into ratio values, as described in previous work [60], [64], [70]. For a given frequency, GAP ratios were computed by dividing the responses to the GAP condition by the mean startle only response. A ratio value close to 0 for the GAP condition indicated healthy gap-detection at a given frequency, while a ratio value close to 1 indicated gap impairment and tinnitus at that frequency. Startle only ratios for each frequency were computed by dividing the responses to the startle condition by the mean startle only response.

Following intense tone exposure, we determined which rats were tinnitus^(+)^ or tinnitus^(−)^. First, outlier responses to the GAP or startle only conditions were removed, which has been done by others to eliminate extreme startle variability [58], [66]. We defined outliers as any responses greater than two standard deviations above the mean. Second, to assess each rat for tinnitus, we pooled the GAP ratios from four out of five baseline gap-detection tests and compared them to four out of the last five gap-detection tests preceding MWM and EPM testing. The worst test out of the five (determined by the highest GAP ratios) was excluded to minimize the chance of any one test inflating gap ratios. Eliminating outliers and the worst test helps minimize false tinnitus positive outcomes. A rat was considered to have tinnitus if it met two criteria: 1) post-exposure GAP ratios were significantly higher than pre-exposure ratios; 2) post-exposure GAP ratios were not significantly lower than post-exposure startle only ratios. We used the first criterion to ensure that gap-detection performance significantly worsened following tone exposure. We used the second criterion to verify that rats could not significantly suppress their startle reflexes in response to the silent gap. Validating gap impairment in this manner helped reduce the possibility that hearing loss, stress, or some other factor significantly worsened but did not genuinely impair gap-detection. Processing the data using these steps reduced data variability, maintained objectivity, and provided a stable behavioral profile for each rat to facilitate proper diagnosis.

Tone-exposed rats not meeting the criteria were placed into the tinnitus^(−)^ group. Another 7 rats underwent pseudo-tone exposure and served as sham controls, but were also individually analyzed. For each group, mean pre-exposure GAP ratios were compared with mean post-exposure GAP ratios to verify any changes induced by the intense tone or pseudo-tone exposure. Additionally, we assessed any changes in startle amplitude by comparing startle force in response to the startle only condition between pre- and post-exposure time points. Decreased startle amplitude could artificially raise GAP ratios and compromise the tinnitus diagnosis [58], [66], whereas increased startle amplitude would indicate hyperacusis-like behavior [80]–[82]. The same 4 out of 5 pre-exposure and post-exposure tests used to evaluate behavior for each rat were pooled for group-wise analysis.

PPI data were analyzed in the same manner as gap-detection data and provided a general assessment of auditory detection and startle stimulus responsiveness. Healthy rats would have reduced their startle reflexes in response to the PPI condition and generated a ratio significantly lower than 1. ABR thresholds were used to evaluate hearing loss by comparing pre-exposure, post-exposure week 1, and post-exposure week 8 recordings within each group, and between the three groups (tinnitus^(+)^, tinnitus^(−)^ and control) at each time point. To determine if there was any relationship between hearing loss and tinnitus, correlation analysis was conducted between post-exposure week 8 left ear ABR thresholds and post-exposure GAP ratios for each group. The post-exposure GAP ratios were averaged at each frequency for each rat from the same 4 out of 5 tests used for tinnitus diagnosis.

MWM data were compared between the three groups to examine the effects of acoustic trauma and tinnitus on spatial learning and memory. Spatial learning was evaluated by the escape latency trials, whereas spatial memory was gauged in the probe trial by the amount of time spent and entries into the target zone. Longer escape latencies and lower affinity for the target zone would suggest impaired spatial learning and memory.

Anxiety level was determined in the EPM by the percent of entries into and time spent in the open-arm. Compared to controls, reduced entries and time in the open-arm would indicate higher anxiety, while increased entries and time suggests less anxiety. Individual rats were also placed into high and low anxiety groups by ranking the percent of open arm time for all animals and dividing the data into quartiles [83]. Rats in the lower quartile below the median were characterized as having high anxiety (HA), while those in the upper quartile above the median were characterized as having low anxiety (LA). The rationale for this analysis was that not all tinnitus patients suffer from anxiety [44], [46], [84]. Therefore, certain tinnitus^(+)^ rats may or may not have anxiety, which would be missed if only group-wise analysis was performed.

For all pre- and post-exposure and between-group comparisons, one-way ANOVA was performed with a post-hoc Bonferroni test to adjust alpha values. For pre- and post-exposure comparisons in gap-detection and PPI data for individual rats, t-test assuming unequal variances was used. Pearson correlation analysis was used to assess the correlation between ABR thresholds and GAP ratios. Statistics such as chi-square were not performed on the HA and LA data from EPM testing due to small sample size. All P values were set to 0.05.

## Results

### Gap Detection and Prepulse Inhibition

**Figure 1:**
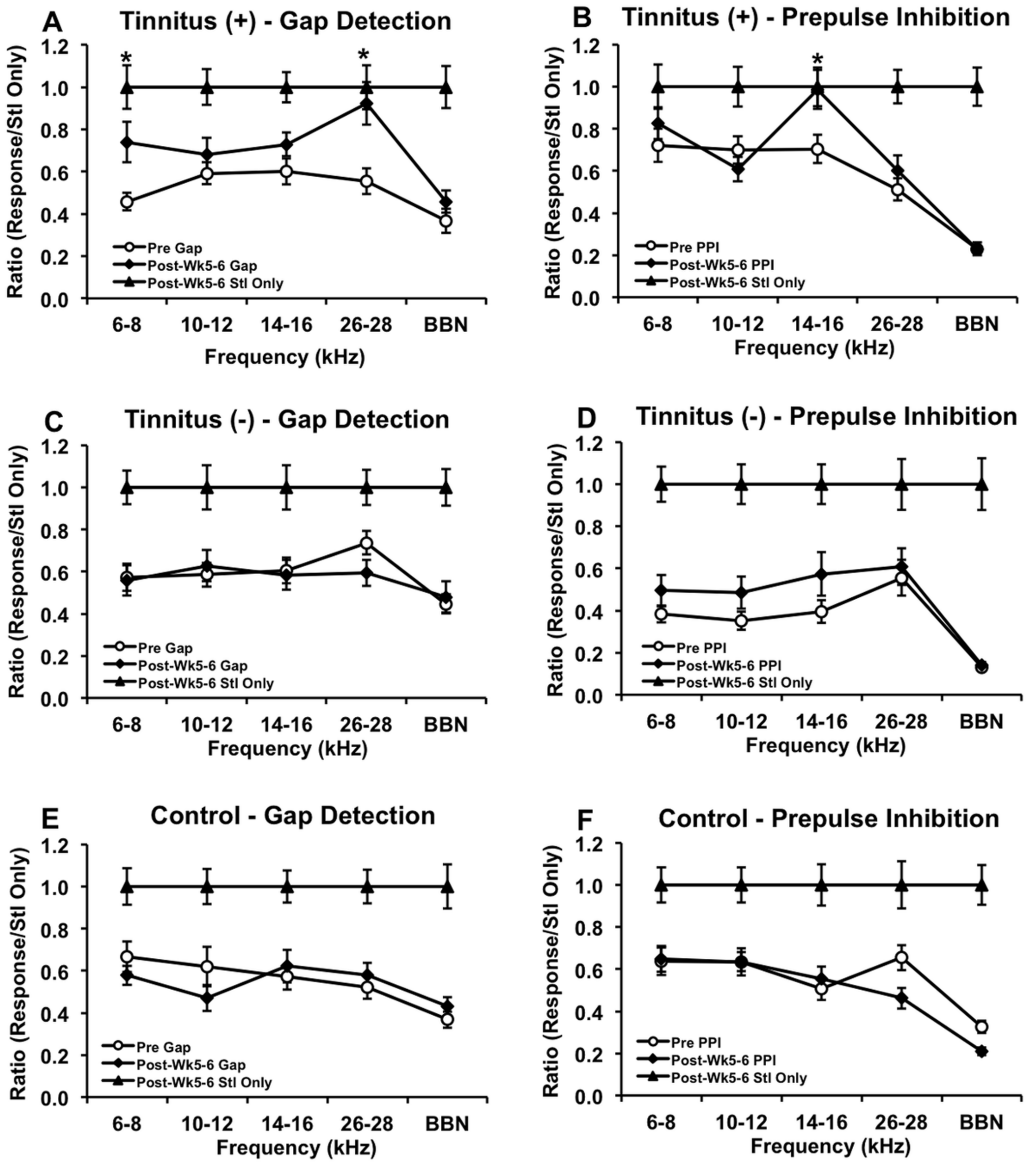
GAP, PPI, and startle only ratios from a representative tinnitus^(+)^, tinnitus^(^^−)^ and control rat. Gap-detection data showed tinnitus at 6–8 and 26–28 kHz in the tinnitus^(+)^ rat (A), which was unaccompanied by same-frequency impairment in PPI (B), although PPI showed auditory detection impairment at 14–16 kHz. Neither the tinnitus^(−)^ (C–D) nor the control rat (E–F) demonstrated tinnitus or auditory detection deficits. Error bars represent the standard error of the mean (SEM). * indicates p<0.05 between pre- and post-GAP, and p>0.05 between post-GAP and post-Stl-Only.

To determine whether a rat had tinnitus, we compared GAP ratios between baseline tests and 5 to 6 weeks following the second noise exposure. Twelve out of eighteen rats met tinnitus criteria: that is, at a given frequency, they exhibited post-exposure GAP ratios that were both significantly higher than pre-exposure GAP ratios and were not significantly lower than post-exposure startle only ratios. PPI ratios were assessed the same way and revealed that the GAP impairments were not accompanied by PPI impairments at the same frequencies. These rats were placed into the tinnitus^(+)^ group, while the other six noise-exposed rats were placed into the tinnitus^(−)^ group. Gap-detection and PPI data from a representative tinnitus^(+)^ (Fig. 1A–B), tinnitus^(−)^ (Fig. 1C–D), and control rat (Fig. 1E–F) are shown. As can be seen, the tinnitus^(+)^ rat had tinnitus at 6–8 kHz [Pre vs. Post GAP (*t* [42] = 2.02, p = 0.011), Post GAP vs. Post Startle Only (*t* [61] = 2.00, p = 0.072)] and 26–28 kHz [Pre vs. Post GAP (*t* [49] = 2.01, p = 0.003), Post GAP vs. Post Startle Only (*t* [59] = 2.00, p = 0.560)]. Post-exposure PPI ratios were not elevated at 6–8 kHz [Pre vs. Post PPI (*t* [58] = 2.00, p = 1.000)] or 26–28 kHz [Pre vs. Post PPI (*t* [57] = 2.00, p = 0.318)], although PPI was impaired at 14–16 kHz [Pre vs. Post PPI (*t* [55] = 2.00, p = 0.016), Post PPI vs. Post Stl Only (*t* [61] = 2.00, p = 0.920)]. The tinnitus^(−)^ and control rats, on the other hand, had no significant impairment in post-exposure GAP or PPI ratios.

**Figure 2:**
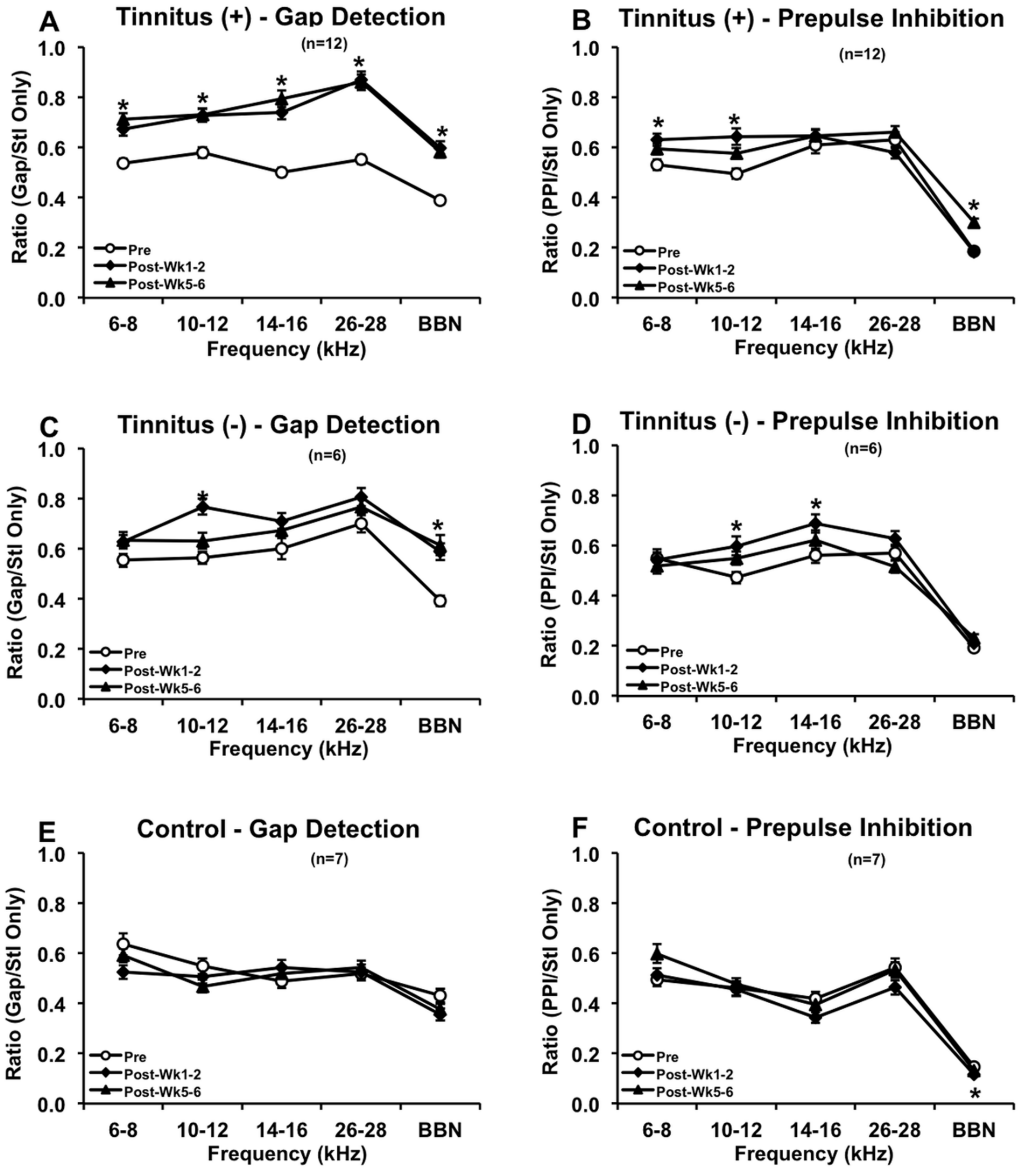
GAP and PPI ratios for the tinnitus^(+)^, tinnitus^(^^−)^ and control groups. Gap-detection data showed tinnitus across all frequency bands and BBN at 1 to 2 and 5 to 6 weeks post-exposure in the tinnitus^(+)^ group (A). PPI data showed auditory detection impairment at 6–8 and 10–12 kHz at 1 to 2 weeks post-exposure, indicating that 1 to 2 week gap impairments at these frequencies may not be specifically due to tinnitus (B). PPI, however, recovered by 5 to 6 weeks post-exposure, except for BBN, which may indicate sensitivity at BBN PPI. The tinnitus^(−)^ group exhibited 10–12 kHz gap impairment at 1 to 2 weeks post-exposure and BBN impairment at 1 to 2 and 5 to 6 weeks (C). PPI, however, also showed 10–12 kHz impairment at 1 to 2 weeks, negating the alleged 10–12 kHz tinnitus (D). The BBN GAP impairment, on the other hand, may be due to hearing loss, since the individual frequency bands were not impaired for the tinnitus^(−)^ group yet their BBN impairment matched that of the tinnitus^(+)^ group, which has similarly elevated hearing thresholds (see Figure 4). No tinnitus or auditory detection impairments were seen in the control group (E–F), although a decrease in BBN PPI ratio was observed, which again may indicate sensitivity changes at this parameter. Error bars represent SEM. * indicates p<0.05.

Grouped GAP and PPI data are depicted in Fig. 2. At 1 to 2 weeks after tone exposure, tinnitus^(+)^ rats showed significant elevations in GAP ratios at 6–8 (*F_(2,1104)_ = *15.043, p<0.001), 10–12 (*F_(2,1111)_ = *11.911, p<0.001), 14–16 (*F_(2,1114)_ = *31.269, p<0.001), and 26–28 kHz (*F_(2,1114)_ = *41.108, p<0.001), and BBN (*F_(2,1111)_ = *29.256, p<0.001) compared to pre-exposure ratio values (ANOVA and *post-hoc* Bonferroni tests; Fig. 2A). Elevated PPI ratios were also seen at post-exposure weeks 1 to 2, including 6–8 (*F_(2,1104)_ = *4.884, p = 0.006) and 10–12 kHz (*F_(2,1105)_ = *8.285, p<0.001; ANOVA and *post-hoc* Bonferroni tests; Fig. 2B). Tinnitus^(−)^ rats demonstrated a significant elevation in Gap ratios at 10–12 kHz (*F_(2,556)_ = *12.531, p<0.001) and BBN (*F_(2,547)_ = *13.277, p<0.001), but also demonstrated elevated PPI ratios at 10–12 (*F_(2,1111)_ = *4.678, p<0.008) and 14–16 kHz (*F_(2,1114)_ = *3.154, p<0.037; ANOVA and *post-hoc* Bonferroni tests; Fig. 2C–D). Although the 10–12 kHz Gap ratio was elevated, the simultaneous elevation at 10–12 kHz PPI suggested that this was due to impairment in auditory detection, as opposed to tinnitus perception. The elevation in the BBN Gap ratio may be due to hearing loss, since the other Gap ratios were not elevated and since the BBN Gap elevation is consistent between tinnitus^(−)^ and tinnitus^(+)^ groups. Control rats showed no differences except for a significant decrease in the BBN PPI ratio (*F_(2,628)_ = *3.221, p = 0.034; ANOVA and *post-hoc* Bonferroni tests; Fig. 2E–F). This may suggest a high degree of variability and/or sensitivity in responses to BBN PPI.

Five to six weeks after intense tone exposure, tinnitus^(+)^ rats retained GAP elevations at 6–8 (*F_(2,1104)_ = *15.043, p<0.001), 10–12 (*F_(2,1111)_ = *11.911, p<0.001), 14–16 (*F_(2,1114)_ = *31.269, p<0.001), and 26–28 kHz (*F_(2,1114)_ = *41.108, p<0.001), and BBN (*F_(2,1111)_ = *29.256, p<0.001, and an elevation at BBN PPI (*F_(2,1078)_ = *38.572, p<0.001; ANOVA and *post-hoc* Bonferroni tests; Fig. 2A–B). Tinnitus^(−)^ rats only showed an elevation at BBN GAP (*F_(2,547)_ = *13.277, p<0.001; ANOVA and *post-hoc* Bonferroni tests; Fig. 2C). The control group did not show any elevations for GAP or PPI (Fig. 2D–F).

**Figure 3:**
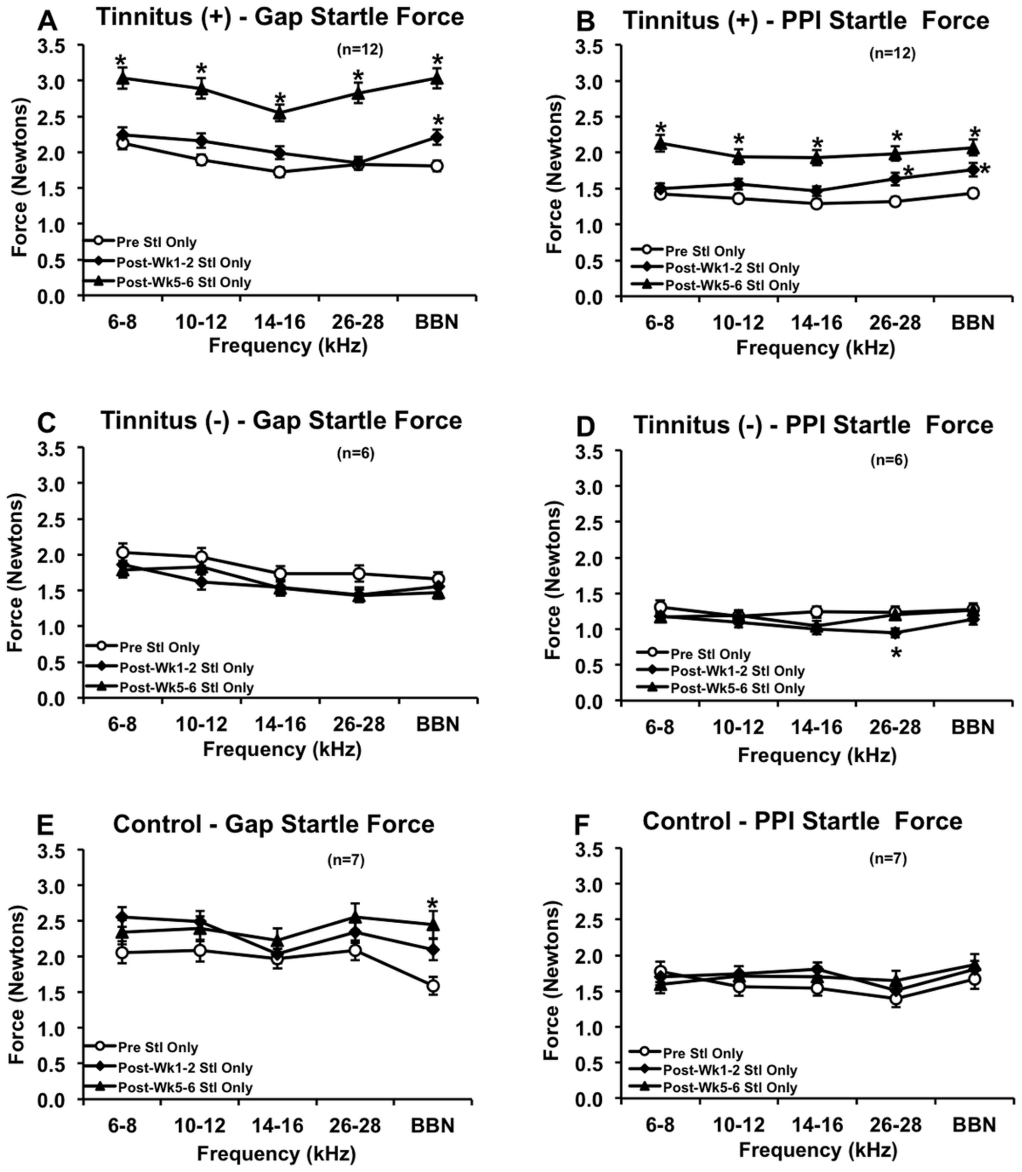
Startle force for the tinnitus^(+)^, tinnitus^(^^−)^ and control groups. The tinnitus^(+)^ group only demonstrated enhanced startle force during BBN background noise at 1 to 2 weeks post-exposure, but showed a dramatic increase during all carrier bands at 5 to 6 weeks (A). Enhanced startle force without background noise was also seen to a small extent at 1 to 2 weeks post-exposure, but to a much greater extent at 5 to 6 weeks (B). The tinnitus^(−)^ group demonstrated no startle force changes (C–D) except for a decrease at 1 to 2 weeks near 26–28 kHz PPI (C–D). The control group showed no startle force changes (E–F) except for an increase at 5–6 weeks during BBN noise (E–F). The tinnitus^(+)^ group by far showed the greatest change in startle force, suggesting hyperacusis-like behavior. All groups showed a sensitization to startle force during background noise, as evidenced by stronger startle force during gap-detection testing (background noise present) compared to PPI testing (background noise absent). For PPI tests, all startle only conditions were identical and were organized by the closest frequency of prepulse incidence to maintain similar comparison to gap-detection. Error bars represent SEM. * indicates p<0.05.

In addition to ratio data, we assessed the effect of noise and pseudo noise exposure on startle force in response to the startle only conditions of the gap-detection and PPI tests (Fig. 3). We found that the tinnitus^(+)^ group exhibited a significant increase in startle force at 1 to 2 weeks post-exposure during BBN background noise (*F_(2,1112)_ = *30.555, p = 0.036) and near 26–28 kHz PPI (*F_(2,1112)_ = *15.713, p = 0.030) and BBN PPI (*F_(2,1105)_ = *12.169, p = 0.035; ANOVA and *post-hoc* Bonferroni tests; Fig. 3A–B). A more dramatic increase in startle force was seen at 5 to 6 weeks post-exposure during all background noise [6–8 (*F_(2,1120)_ = *17.723, p<0.001), 10–12 (*F_(2,1130)_ = *21.576, p<0.001), 14–16 (*F_(2,1129)_ = *20.097, p<0.001), and 26–28 kHz (*F_(2,1121)_ = *29.638, p<0.001), and BBN (*F_(2,1112)_ = *30.555, p<0.001)] and near-PPI conditions [6–8 (*F_(2,1106)_ = *19.633, p<0.001), 10–12 (*F_(2,1116)_ = *13.236, p<0.001), 14–16 (*F_(2,1106)_ = *17.338, p<0.001), and 26–28 kHz (*F_(2,1112)_ = *15.713, p<0.001), and BBN (*F_(2,1105)_ = *12.169, p<0.001)], suggesting hyperacusis-like behavior (ANOVA and *post-hoc* Bonferroni tests; Fig. 3A–B). The tinnitus^(−)^ group, however, showed no changes in startle force except for a decrease near 26–28 kHz PPI at 1 to 2 weeks post-exposure (*F_(2,557)_ = *4.364, p = 0.021), which may have been due to hearing loss (ANOVA and *post-hoc* Bonferroni tests; Fig. 3C–D). The control group also showed no changes in startle force except for an increase during BBN background noise at 5 to 6 weeks post-exposure (*F_(2,630)_ = *7.355, p<0.001; ANOVA and *post-hoc* Bonferroni tests; Fig. 3E–F). Overall, our results indicate that unilateral noise exposure may not always reduce startle force and can actually increase it.

### ABR Thresholds

**Figure 4:**
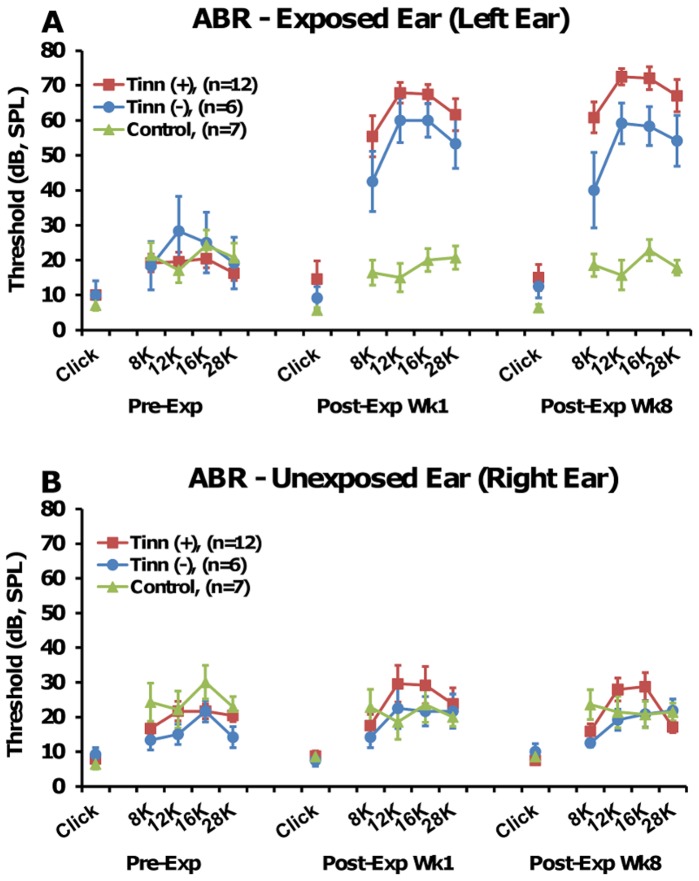
Auditory brainstem responses from the exposed left ear (A) and unexposed right ear (B). In the exposed ear (A), both the tinnitus^(+)^ and tinnitus^(−)^ groups showed significant threshold shifts across tone-burst frequencies at 1 and 8 weeks post-exposure, with the strongest elevations occurring at 12 and 16 kHz. Overall, the tinnitus^(+)^ group had significantly higher hearing thresholds than the tinnitus^(−)^ group, although the thresholds were not significantly higher at any individual frequency or click. (B) No significant threshold shifts were seen in the unexposed ear for tinnitus^(+)^ and tinnitus^(−)^ groups. The control group showed no changes in either ear (A–B). Error bars represent SEM.

ABR responses were recorded before tone exposure and at 1 and 8 weeks post-exposure to measure the effects of acoustic trauma on hearing thresholds (Fig. 4). While click was unaffected in either group, thresholds were significantly elevated across tone burst frequencies in the exposed ear of the tinnitus^(+)^ group at 8 (*F_(2,33)_ = *25.546, p<0.001), 12 (*F_(2,33)_ = *120.667, p<0.001), 16 (*F_(2,33)_* 96.083, p<0.001), and 28 kHz (*F_(2,33)_ = *49.811, p<0.001) and the tinnitus^(−)^ group at 12 (*F_(2,15)_ = *5.687, p = 0.029), 16 (*F_(2,15)_ = *9.034, p = 0.006), and 28 kHz (*F_(2,15)_ = *7.678, p = 0.013; ANOVA and *post-hoc* Bonferroni tests; Fig. 4A). Recordings collected at post-exposure week 8 indicated that after several weeks, hearing thresholds remained elevated in both the tinnitus^(+)^ group at 8 (*F_(2,33)_ = *25.546, p<0.001), 12 (*F_(2,33)_ = *120.667, <0.001), 16 (*F_(2,33)_ = *96.083, p<0.001), and 28 kHz (*F_(2,33)_ = *49.811, p<0.001) and in the tinnitus^(−)^ group at 12 (*F_(2,15)_ = *5.687, p = 0.034), 16 (*F_(2,15)_ = *9.034, p = 0.008), and 28 kHz (*F_(2,15)_ = *7.678, p = 0.011; ANOVA and *post-hoc* Bonferroni tests; Fig. 4A). No significant differences between time points were observed in the unexposed ear of the tinnitus^(+)^ and tinnitus^(−)^ groups (Fig. 4B), and no elevations were seen in either ear of the control group.

Thresholds were also compared between the 3 groups at each time point before and after tone exposure. This allowed us to determine whether there was a pre-existing or induced difference in hearing between groups, which could become a confound when interpreting how tinnitus and hyperacusis-like behavior affected learning, memory and anxiety. When compared to the control group, thresholds collected from the exposed ear at post-exposure week 1 were overall significantly elevated in the tinnitus^(+)^ group (*F_(2,122)_ = *34.711, p<0.001). Specifically, elevations were found at 8 (*F_(2,22)_ = *10.146, p = 0.001), 12 (*F_(2,22)_ = *47.273, p<0.001), 16 (*F_(2,22)_ = *52.754, p<0.001), and 28 kHz (*F_(2,22)_ = *17.759, p<0.001). The tinnitus^(−)^ group also showed overall elevated thresholds (*F_(2,122)_ = *34.711, p<0.001), specifically at 12 (*F_(2,22)_ = *47.273, p<0.001), 16 (*F_(2,22)_ = *52.754, p<0.001), and 28 kHz (*F_(2,22)_ = *17.759, p = 0.002; ANOVA and *post-hoc* Bonferroni tests; Fig. 4A). Thresholds remained elevated overall at post-exposure week 8 in the tinnitus^(+)^ group (*F_(2,122)_ = *40.256, p<0.001), specifically at 8 (*F_(2,22)_* 13.520, p<0.001), 12 (*F_(2,22)_ = *63.444, p<0.001), 16 (*F_(2,22)_ = *43.218, p<0.001), and 28 kHz (*F_(2,22)_ = *25.711, p<0.001) and in the tinnitus^(−)^ group (*F_(2,122)_ = *40.256, p<0.001), specifically at 12 (*F_(2,22)_ = *63.444, p<0.001), 16 (*F_(2,22)_ = *43.218, p<0.001), and 28 kHz (*F_(2,22)_ = *25.711, p = 0.001; ANOVA and *post-hoc* Bonferroni tests; Fig. 4A).

**Figure 5:**
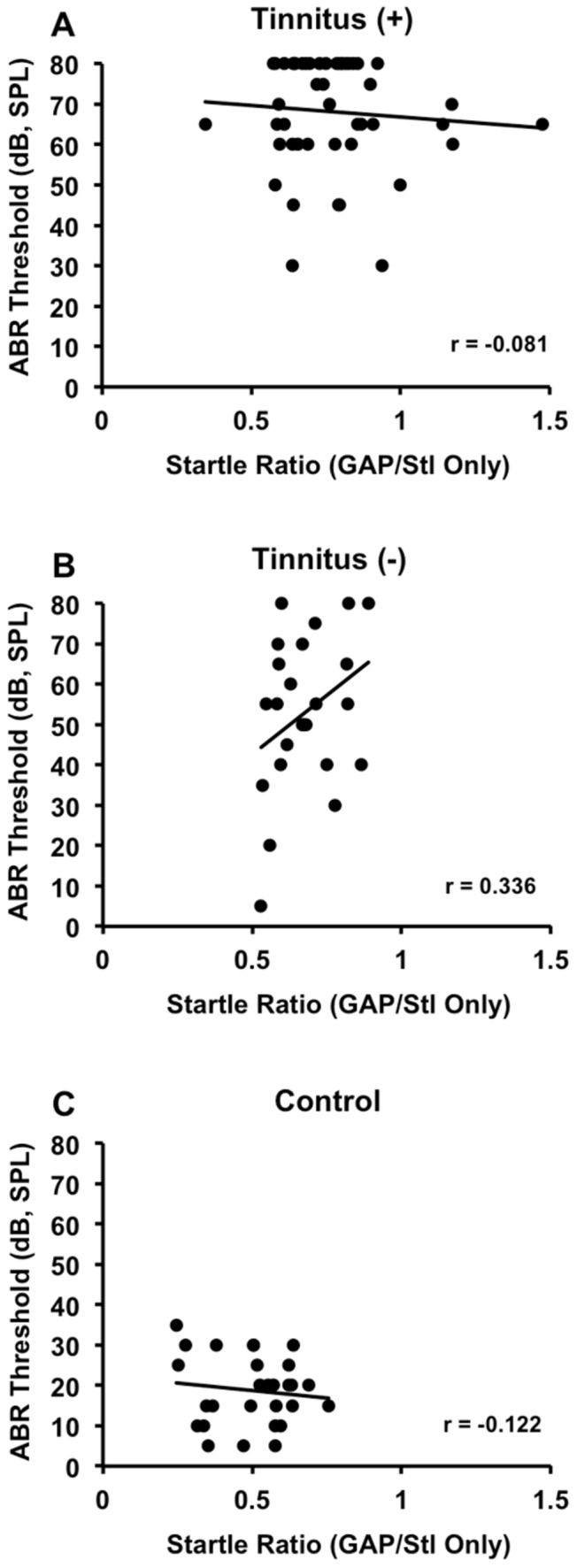
Correlation between ABR thresholds and GAP ratios for tinnitus^(+)^ (A), tinnitus^(−)^ (B), and control (C) groups. No groups exhibited a significant correlation, suggesting that although tinnitus^(+)^ rats had more overall hearing loss (see Figure 4), it was not the only factor accounting for elevated GAP ratios and thus behavioral manifestation of tinnitus.

We also compared thresholds between the tinnitus^(+)^ and tinnitus^(−)^ group. While there was no difference between these two groups at 1 week post-exposure (*F_(2,122)_ = *34.711, p = 0.251), the tinnitus^(+)^ group showed overall higher thresholds than the tinnitus^(−)^ group at 8 weeks post-exposure (*F_(2,122)_ = *40.256, p<0.030). Although there were no significant differences between these two groups in clicks or individual frequencies, that may be due to lower sample size and less statistical power compared to combined-frequency analysis. To further explore the relationship between hearing loss and tinnitus, we conducted correlation analysis between ABR thresholds and GAP ratios for each group. We found that thresholds and ratios were not significantly correlated for the tinnitus^(+)^ (r = −0.081, p = 0.586), tinnitus^(−)^ (r = 0.336, p = 0.109), or control group (r = −0.122, p = 0.536) (Fig. 5A–C), suggesting that although hearing loss may have been greater overall in the tinnitus^(+)^ group, it was not specifically linked to elevated GAP ratios.

### Morris Water Maze (MWM)

**Figure 6:**
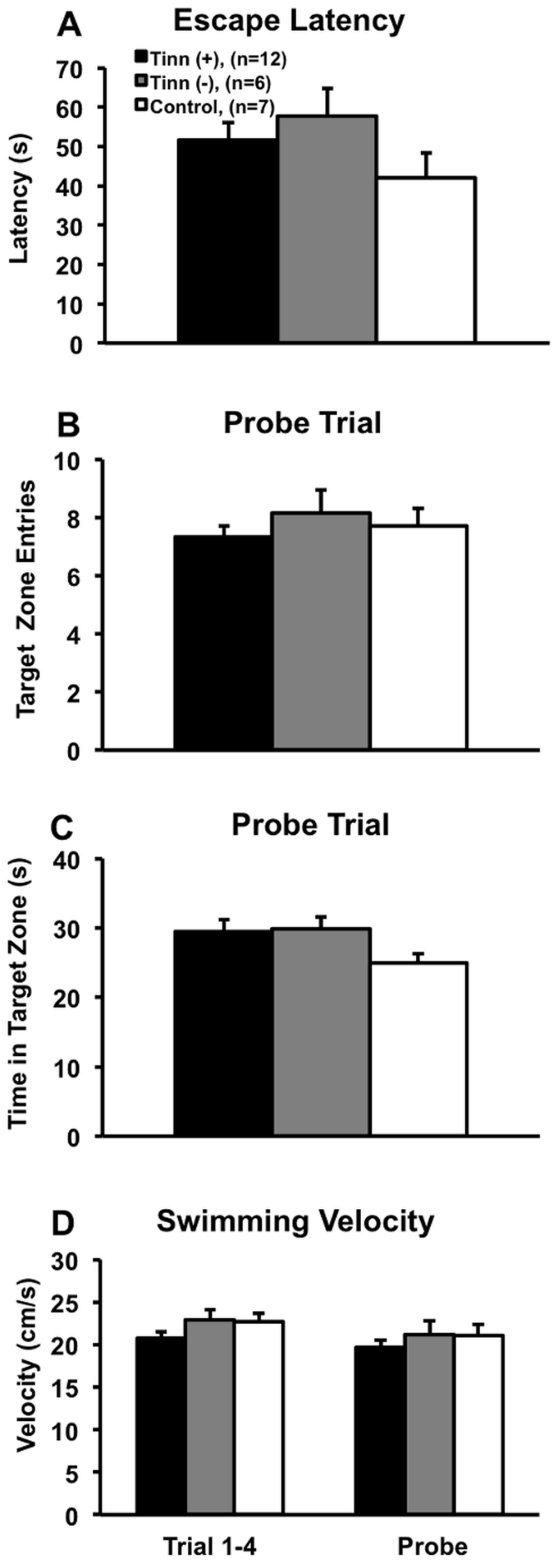
Morris water maze escape latency and probe trial data. No significant differences were seen between the tinnitus^(+)^, tinnitus^(−)^ and control groups in escape latency (A), probe trial target zone entries (B), probe trial target zone time (C), and velocity (D). This indicated similar spatial learning and memory across groups. Error bars represent SEM.

Tinnitus^(+)^ and tinnitus^(−)^ rats underwent a one-day MWM procedure as described elsewhere [79] 6 weeks after tone exposure to assess the effects of tinnitus, hearing loss, and hyperacusis-like behavior on spatial learning and memory (Fig. 6). No significant differences were found between any of the three groups, including tinnitus^(+)^ and tinnitus^(−)^ (*F_(2,97)_ = *1.498, p = 1.000), tinnitus^(+)^ and control (*F_(2,97)_ = *1.498, p = 0.687), and tinnitus^(−)^ and control (*F_(2,97)_ = *1.498, p = 0.280; ANOVA and *post-hoc* Bonferroni tests; Fig. 6A). Although rats only underwent 4 escape latency trials, the mean escape latencies of their last 2 trials (when learning had been established) were similar to the last 4 trials of Sprague-Dawley control rats from our unpublished study using a one-day, 12 trial procedure (data not shown), which has been successfully used in other studies to substantiate strong spatial learning and memory [85], [86]. In addition, Long-Evans rats tend to have superior spatial cognition compared to many other domesticated strains [87]. Taken together, this justified our one-day MWM protocol.

Probe trial testing followed trial 4 of escape latency testing. There were no significant differences between groups in target zone entries, including tinnitus^(+)^ and tinnitus^(−)^ (*F_(2,22)_ = *0.588, p = 0.883), tinnitus^(+)^ and control (*F_(2,22)_* 0.588, p = 1.000), and tinnitus^(−)^ and control (*F_(2,22)_ = *0.588, p = 1.000; ANOVA and *post-hoc* Bonferroni tests; Fig. 6B). There were also no significant differences in target zone time between tinnitus^(+)^ and tinnitus^(−)^ (*F_(2,22)_ = *1.999, p = 1.000), tinnitus^(+)^ and control (*F_(2,22)_ = *1.999, p = 0.256), or tinnitus^(−)^ and control groups (*F_(2,22)_ = *1.999, p = 0.308; ANOVA and *post-hoc* Bonferroni tests; Fig. 6C). Finally, there were no significant differences in swimming velocity between tinnitus^(+)^ and tinnitus^(−)^ (*F_(2,97)_ = *0.586, p = 1.000), tinnitus^(+)^ and control (*F_(2,97)_ = *0.586, p = 1.000), or tinnitus^(−)^ and control (*F_(2,97)_ = *0.586, p = 1.000), suggesting similar mobility levels (ANOVA and *post-hoc* Bonferroni tests; Fig. 6D).

### Elevated Plus Maze (EPM)

**Figure 7:**
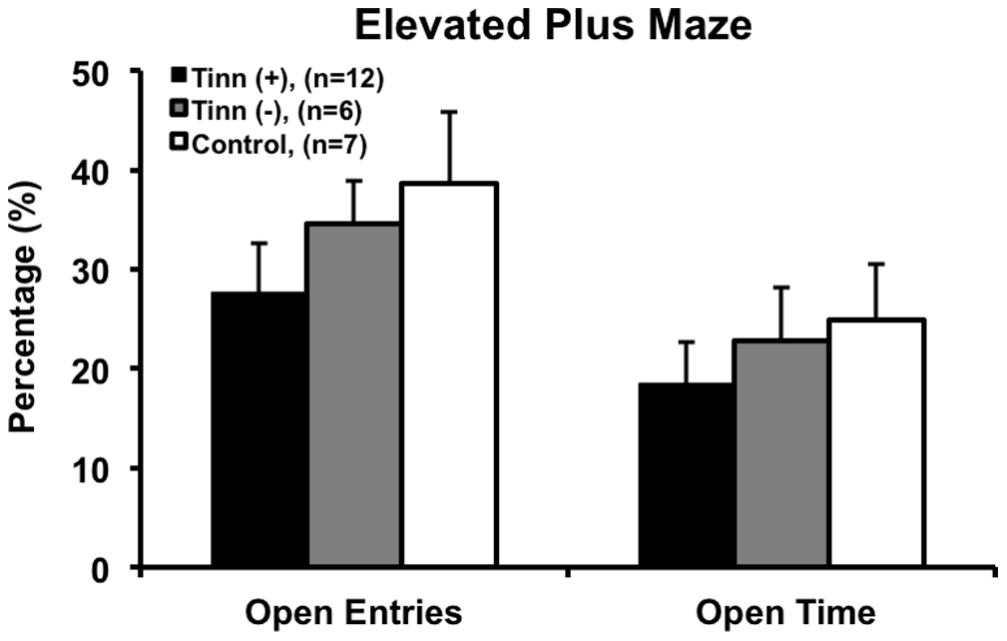
Percent of open-arm entries and open-arm time in the elevated plus maze. No significant differences were seen between the tinnitus^(+)^, tinnitus^(−)^, and control groups in percent of open-arm entries or percent of open-arm time, indicating similar anxiety level across groups. Error bars represent SEM.

EPM was conducted to explore the effects of intense tone-induced tinnitus, hearing loss, and hyperacusis-like behavior on anxiety (Figure 7). No significant differences were observed between groups on percent of open-arm entries, including tinnitus^(+)^ and tinnitus^(−)^ (*F_(2,22)_ = *1.049, p = 1.000), tinnitus^(+)^ and control (*F_(2, 22)_ = *1.049, p = 0.525), and tinnitus^(−)^ and control (*F_(2,22)_ = *1.049, p = 1.000; ANOVA and *post-hoc* Bonferroni tests; Fig. 7). Furthermore, no differences were found between groups on percent of open-arm time, including tinnitus^(+)^ and tinnitus^(−)^ (*F_(2,22)_ = *0.479, p = 1.000), tinnitus^(+)^ and control (*F_(2,22)_ = *0.479, p = 1.000), and tinnitus^(−)^ and control (*F_(2,22)_ = *0.479, p = 1.000; ANOVA and *post-hoc* Bonferroni tests; Fig. 7).

In addition to group-wise analysis, we investigated whether individual rats had high anxiety (HA) or low anxiety (LA). For the tinnitus^(+)^ group, 4 rats had HA and 1 had LA, while for the tinnitus^(−)^ group, no rats had HA and 1 had LA. For the control group, 1 rat had HA and 2 had LA. HA rats had a lower number of open-arm entries and a similar number of closed-arm entries compared to LA rats (not shown), indicating that the groups truly differed on anxiety level. Although sample sizes in the HA and LA groups were small, these results support clinical findings by showing that tinnitus and hyperacusis may result in a greater likelihood for comorbid anxiety, but that not all affected individuals experience anxiety.

## Discussion

### Noise-induced Tinnitus and Individualized Diagnosis

We found that two exposures to a 10 kHz tone at 118–120 dB SPL and 2–3 hours duration (2 hours for first exposure, 3 hours for the second) induced noise-like tinnitus for at least 6 weeks in twelve out of eighteen rats. First, the current study may be clinically relevant to patients who have sustained acoustic trauma on multiple occasions, or on a regular basis as a result of occupational hazard [2], [88]–[90]. Second, our findings are corroborated by other reports showing that exposure to tones in the range of 10 to 17 kHz can produce tinnitus from anywhere between 6 to 32 kHz [20], [33], [54], [73], [91]. A recent study by our lab demonstrated that single blast exposure (14 psi, 10 ms pulse duration) induced immediate noise-like tinnitus, which later shifted towards high-frequencies [60]. Overall, experimental findings are in line with human data, where individuals have been exposed to acoustic trauma with a range of characteristics and experience tinnitus with varying features [88]–[90], [92], [93].

A key goal of this study was to develop a reliable method to diagnose chronic tinnitus in rats at individual level. This is a critical factor since many studies have either not divided animals into tinnitus positive and negative groups [60], [61], [63], [70], [72], [73], [75], [76] or used a standardized method to do so [20], [64], [66], [67], [71]. Our results demonstrated that taking the best four out of five behavioral tests and removing outliers generated a stable baseline behavioral profile for a rat, as evidenced by the significantly attenuated GAP responses compared to startle only responses (Figure 1). This analytical method provides an objective and reliable way of determining whether and when rats have stable baseline behavior. Determining not only baseline stability but the typical GAP responses for each rat is crucial since baseline GAP values affect whether post-exposure values appear elevated or not, which in turn influences tinnitus diagnosis. Our data also showed that this method generated a stable post-exposure assessment, which is important in achieving accurate diagnosis and avoiding false tinnitus positive results. This is validated by the fact that twelve exposed rats were diagnosed with tinnitus while six were not, and that no control rats were diagnosed with tinnitus. Our results support other reports that noise exposure does not induce tinnitus in every subject [20], [64]–[67], [71], [94].

Another finding from individualized analysis was that different rats developed tinnitus at different frequencies. Specifically, we found that tinnitus ranged from 8 to 28 kHz. This is in line with another study where intense tone exposure (12 kHz, 126 dB, SPL, 2 h) induced tinnitus between 6–24 kHz [20]. In such cases, maladaptive neuroplastic changes giving rise to tinnitus perception may vary across animals over time and account for the disparity in pitch. Identifying the frequencies of tinnitus in specific rats may play an important role in pinpointing correlates of tinnitus, such as latency and amplitude changes in ABR waves, and changes in spontaneous activity, bursting, and synchrony in tonotopically-organized structures such as the dorsal cochlear nucleus, inferior colliculus, and auditory cortex. Relying solely on group-wise analysis may obscure certain frequencies of tinnitus manifestation and fail to yield the most accurate profile of tinnitus induction.

Our tinnitus^(+)^ rats exhibited higher post-exposure hearing thresholds than tinnitus^(−)^ or control rats, suggesting that hearing loss plays a role in tinnitus manifestation. The lack of significant correlation between hearing thresholds and GAP ratios, however, indicates that the currently used diagnostic method adequately revealed tinnitus manifestation in the tinnitus^(+)^ rats.

### Influence of Tinnitus and Hearing Loss on Hyperacusis

In addition to behavioral evidence of tinnitus, the tinnitus^(+)^ group showed some significantly increased startle response amplitudes to the startle only conditions at 1 to 2 weeks after tone exposure, followed by more substantial increases at 5 to 6 weeks post-exposure. This is suggestive of hyperacusis-like behavior in the tinnitus^(+)^ group and is supported by similar findings [80], [81], though only Chen and colleagues tested for simultaneous tinnitus. Other studies have found an association between salicylate- and noise-induced tinnitus and stronger than expected prepulse inhibition, which may also be indicative of hyperacusis [74], [95]. A close association between tinnitus and hyperacusis would be expected, since the two are highly correlated in the clinical population [38]–[40] and putatively share similar pathophysiology [81], [96]–[98]. In fact, several studies reporting increased startle amplitude in response to noise exposure, age-related hearing loss, and salicylate injections might have also found tinnitus manifestation if they had tested their subjects [81], [82], [99]–[101]. Future work in animals should consider simultaneous assessment for tinnitus and hyperacusis, so that the relationship can be better studied and understood.

Despite these common findings, others have found a decrease in startle amplitude following noise exposure and/or hearing loss [58], [66], [102]. One reason for the disparity may be the degree and frequencies of the induced hearing loss. That is, partial hearing loss may be necessary to enhance startle amplitude. The present study used 10 kHz tone exposures to induce a transient 6–12 kHz loss in auditory detection and permanent hearing threshold elevations from 8 to 28 kHz, with 12 and 16 kHz sustaining the highest elevations (∼49–57 dB) and 8 kHz sustaining the lowest elevations (∼39–42 dB). Studies finding increased startle responsivity as a result of aging [82], [101] and salicylate injections [99], [100] also reported high-frequency hearing loss, which is theorized to have caused the hyperacusis-like behavior through overrepresentation of low frequencies and increased central auditory system gain. Sun and colleagues (2012) found high-frequency hearing loss immediately following noise exposure (11–13 kHz, 1 hour, 120 dB SPL), which was accompanied by increased startle responsivity to low-frequency stimuli. Rybalko and colleagues, however, found no hearing loss at 3–5 months following noise exposure (BBN, 125 dB SPL, 8 minutes) at postnatal day 14, but found reduction in startle responsivity to all but 2 kHz stimuli. For others that reported reduced startle responsivity after noise exposure, either hearing loss was not assessed [58] or a wide band noise exposure was used [66], which may not have led to overrepresentation of certain frequencies. Therefore, the rats in our study may have developed hyperacusis from hearing loss (12–16 kHz), which resulted in overrepresentation of low frequencies (below 8 kHz) and increased startle responsivity to noise burst stimuli. While the current study did not test startle reflexes in response to low-frequency stimuli, it is likely that lower-frequency responsivity fed into broadband responsivity [102].

### Effect of Tinnitus on Cognition and Anxiety

Tinnitus^(+)^ rats showed no significant differences on escape latency or probe trials compared to tinnitus^(−)^ or control rats. This suggests that there was no significant difference in spatial learning and memory, which is consistent with previous reports [31]. Mixed results have been found in animal studies, with tinnitus affecting some cognitive and behavioral functioning such as impulse control and social interaction [32], [33], but not affecting spatial cognition and serial reaction time accuracy [31], [33]. The inconsistent findings from animal studies match those from clinical studies in that some subjective accounts by patients and experimental evidence suggests that tinnitus interferes with cognitive functioning [8]–[12], [29]. Other accounts and assessments in humans including tests of serial and spatial recall and five-choice serial reaction time with a dual task for memory [10], [29] have found no tinnitus effect. The cognitive-behavioral test paradigms used, as well as the characteristics of the tinnitus perception, may therefore play a significant role in tinnitus assessment. The inconsistent results in humans may also suggest that some cognitive deficits are not caused by the tinnitus itself, but may be due to underlying factors, such as comorbid mood, anxiety, or other psychological disorders.

We did not find any significant differences in anxiety level between the tinnitus^(+)^, tinnitus^(−)^, or control rats using group-wise analysis. These results match other studies that exposed rats to noise (95–110 dB SPL, 1–2 hour duration) and found no effect on EPM performance [33], [35], though only Zheng and colleagues tested for tinnitus and neither group examined the long-term progression of hearing loss or hyperacusis. When examining individual animals, we found that 4 tinnitus^(+)^ rats had high anxiety (HA) and 1 had low anxiety (LA). Among the tinnitus^(−)^ group, no rats had HA and 1 had LA, while in the control group, 1 rat had HA and 2 had LA. Although human studies have reported significantly higher anxiety in tinnitus patients compared to the general population [44], [46], [84], our results agree with these studies in that anxious predisposition is found in some but not all tinnitus patients. More studies with a greater sample size and varying severities of tinnitus are needed to further investigate this relationship. The causative factors within this relationship must also be taken into consideration, given the high comorbidity rate of tinnitus with anxiety, as well as depression [42]–[47]. It remains undetermined whether tinnitus can always cause anxiety and depression, or whether individuals with pre-existing or predispositions to anxiety and depression are more inclined to suffer from tinnitus perception.

As with tinnitus and cognitive functioning, anxiety and emotional deficits may only manifest in tinnitus with certain characteristics and with certain tests. In humans, anxiety can often occur when the affected individual is trying to sleep or at various times during routine activities. Monitoring sleep and using other methods to evaluate the emotional status of rats may help clarify the relationship between tinnitus and anxiety. Examples include measuring insomnia, corticosterone, 5-HIAA level (a metabolite of 5-HT that can reflect serotonergic activity and stress), the light-dark box test, and weight levels. Assessing depression-like behavior with forced-swim and sucrose consumption tests may also yield valuable information.

### Effect of Hyperacusis on Cognition and Anxiety

Since the tinnitus^(+)^ group demonstrated hyperacusis-like behavior but showed no significant cognitive deficits or increased anxiety, it appeared that hyperacusis also had no effect on cognition and anxiety. Like with the negative effect of tinnitus on cognitive-emotional functioning, these results were unexpected since hyperacusis can result in distress, social withdrawal [41], sleeping impairment [103], and increased propensity for depression and anxiety [104]–[106]. Although the relationship between hyperacusis and cognition has not been addressed in previous studies, it is reasonable to project that hyperacusis and its negative side affects would impair cognitive functioning, including learning and memory. Published MRI data have shown that semantic dementia patients with tinnitus or hyperacusis sustain relative preservation of grey matter in the posterior superior temporal lobe and decreased grey matter in the orbitofrontal cortex and medial geniculate nucleus [107], suggesting involvement of the limbic system, which may account for their emotional reactivity to auditory perception. As with tinnitus, however, it may be that other tools besides MWM and EPM should be sought to assess the cognitive-emotional effects of hyperacusis in rats. Additionally, the measurement used to identify hyperacusis may need refining. Since hyperacusis is most often defined as decreased tolerance to moderate, everyday sound, the most convincing evidence of hyperacusis in rats may be a statistically significant startle response to a moderate-intensity startle stimulus (i.e. lower than 80 dB), which has not been reported to date. Clearly, future studies in this area are needed.

## Conclusions

The current study used a statistically-driven method to demonstrate that tinnitus can be reliably identified in individual rats. This was supported by our results showing that noise-exposed rats can be separated into tinnitus^(+)^ and tinnitus^(−)^ groups and that all non-exposed controls tested tinnitus negative. In addition, the tinnitus^(+)^ group demonstrated evidence of hyperacusis-like behavior, which is frequently seen in the clinical population. We found, however, that neither tinnitus, hearing loss, nor hyperacusis yielded a group-wise effect on cognition and anxiety, although the majority of rats with high anxiety came from the tinnitus^(+)^ group. These results, however, are all in line with complex clinical findings, and underscore the difficulties of characterizing non-auditory dysfunction in tinnitus patients and developing treatment methods. The effects of tinnitus on functioning, including difficulties in sleeping and concentrating, irritability, and increased risk for depression, anxiety, and even suicide, all underline the fact that they are among the most important consequences of tinnitus. In order for animal models of tinnitus to achieve the greatest relevance, these factors must be considered, and optimal methods for detecting tinnitus and related auditory and non-auditory functioning, including alternative tests for cognition and anxiety/depression, must be sought.
